# Not every pseudoword disrupts word recognition: an ERP study

**DOI:** 10.1186/1744-9081-2-36

**Published:** 2006-10-24

**Authors:** Claudia K Friedrich, Carsten Eulitz, Aditi Lahiri

**Affiliations:** 1 Department of Linguistics, University of Konstanz, Germany; 2 Biological Psychology and Neuropsychology, University of Hamburg, Germany

## Abstract

**Background:**

If all available acoustic phonetic information of words is used during lexical access and consequently stored in the mental lexicon, then all pseudowords that deviate in a single acoustic feature from a word should hamper word recognition. By contrast, models assuming underspecification of redundant phonological information in the mental lexicon predict a differential disruption of word recognition dependent on the phonological structure of the pseudoword. Using neurophysiological measures, the present study tested the predicted asymmetric disruption by assuming that coronal place of articulation for consonants is redundant.

**Methods:**

Event-related potentials (ERPs) were recorded during a lexical decision task. The focus of interest was on word medial consonants. The crucial pseudowords were created by replacing the place of articulation of the medial consonant in German disyllabic words. We analyzed the differential temporal characteristics of the N400 pseudoword effect.

**Results:**

N400 amplitudes for pseudowords were enhanced compared to words. As the uniqueness and deviation points differ for coronal and non-coronal items, the ERPs had to be correspondingly adjusted. The adjusted ERPs revealed that the N400 pseudoword effect starts earlier for coronal than for non-coronal pseudoword variants. Thus, non-coronal variants are accepted as words longer than the coronal variants.

**Conclusion:**

Our results indicate that lexical representations of words containing medial coronal consonants are initially activated by their corresponding non-coronal pseudowords. The most plausible explanation for the asymmetric neuronal processing of coronal and non-coronal pseudoword variants is an underspecified coronal place of articulation in the mental lexicon.

## Background

Despite the fact that we perceive speech in an apparently involuntary fashion, one requires several processing stages to extract the meaning of an utterance. Acoustic-phonetic features that build up the signal have to be extracted and mapped onto knowledge stored in the listeners' long-term memory in the form of lexical representations [1]. Mental representations must be capable of successfully handling the tremendous amount of variability in the speech signal with which the recognition system is confronted. A frequent source of variation is assimilation where segments take on the properties of other sounds in close proximity. A common assimilation discussed frequently in the literature involves consonants with a CORONAL place of articulation (PLACE; e.g., /t, d, n/), which appear to borrow the PLACE information of the segment that immediately follows. The /n/ in *rain*, for example, can take on the PLACE of the following LABIAL /b/ in *rainbow *resulting in *raimbow (note that here and in the following examples an asterisk marks pseudowords).

Different accounts have been proposed to explore the fact that assimilated *raim does not disrupt recognition of *rain *[2-16]. One of these approaches is the assumption of a featurally underspecified lexicon (FUL) [2-4]. In an underspecified lexicon feature value slots can be left empty particularly for feature values that frequently show variation. Leaving the PLACE slot for coronal feature values empty (underspecified representation) allows activation from systematic variants that are specified for PLACE, as these will not mismatch with the underspecified value in the lexical representation. Thus, *raim can activate the word rain. Invariant feature values, e.g. [LABIAL] and [DORSAL] PLACE, are specified in the lexical representation because they can reliably contribute to word identification. Consider for example the dorsal /K/ in *long days *that will not become a coronal /n/ even if it is followed by a coronal /d/. The extraction of features from the signal, and the presence or absence of those features in the representation of words predicts asymmetric activation patterns. Words are activated if signal and representation contain the same information, or if an extracted feature is not represented in the lexicon. In contrast, words are rejected if signal and representation contain incompatible features.

Alternative approaches to the FUL model strengthen the importance of context for the correct recognition of assimilated forms. Gaskell and Marslen-Wilson assume that mapping speech onto lexical representations involves on-line phonological inference that detects systematic variation [5,6]. In this approach it is proposed that listeners cope with assimilation by inverting phonological rules in a given context. Accordingly, when presented with *raimbow*, listeners infer that a labial followed by another labial may be an underlying coronal. Evidence for contextual sensitivity in the analysis of feature changes has been provided in cross-modal priming experiments [5-7]. Furthermore, a combined impact of context and phonetic detail has been proposed by Gow [8-10]. He argues that assimilation does not change a feature completely, but provides information about both the underlying form of the assimilated segment and the surface form of the following segment. According to this point of view listeners detect assimilated features, use this information to anticipate the upcoming segment, and align the assimilated feature to this subsequent element. Indeed, some empirical evidence suggests that context in combination with phonetic detail drives compensation for assimilation [8-12].

However, the representational-cum-mapping hypothesis of FUL also received support from psycholinguistic as well as from neurolinguistic studies. Behavioral priming experiments showed that only non-coronal variants (e.g., *wickib) activate associates of coronal words (e.g., *wicked*), whereas coronal variants (e.g., *sanctun) do not activate associates of non-coronal words (e.g., *sanctum*) [4]. Moreover, recent cross-modal priming studies challenged the impact of context by showing that lexical activation of underlying coronal entries appears to be equally tolerant to contextually appropriate and inappropriate changes of coronal elements [13,14]. Finally, mismatch negativity (MMN) observed in event related brain potentials (ERPs) provides evidence for FUL. MMN is elicited by infrequent deviant stimuli that are presented after a random number of frequent standard stimuli (see [17] for an overview). Earlier latency and higher amplitude MMN values were found for coronal deviants among non-coronal standards, as compared to non-coronal deviants among coronal standards. This suggests that the representations activated by the coronal standards do not have PLACE specified [18].

Psycholinguistic research on underspecified entries largely looked at assimilation contexts occurring in word final position. Here one could argue that all assimilation variants are stored, since they occur in predictable environments and will be part of the listener's experience. However, if lexical underspecification is part of the mental representation, then asymmetric activation should also occur for other positions within a word. Despite the fact that variants in word medial position can never occur due to the context of another word FUL expects asymmetric acceptance: a labial or dorsal variant of an underlying underspecified coronal would be tolerated but not vice versa. For example, *wimmer will be accepted as a variant of *winner*, but *tunny is an impossible variant of the word *tummy*. Such an asymmetry also affects the rejection of alternates as possible variants. The prediction would be that variants like *tunny should be rejected as a word since they conflict immediately with *tummy*. In contrast, it is possible that *wimmer is more difficult to reject since it is acceptable as a variant of the real word *winner*.

In the present experiment we tested the neurophysiological validity of an underspecified lexicon with underspecified word medial coronal consonants, utilizing the high temporal resolution of ERPs recorded in a lexical decision task. Previous ERP research suggests that a specific negative ERP component, the N400, is sensitive to the time-course of cognitive processes underlying word recognition. Since its first description [19], the N400 has not only been correlated to aspects of semantic processing in sentence and priming contexts, but also to single word processing (see [20] for a review). The N400 pseudoword effect is characterized by larger and longer lasting negative amplitudes for isolated spoken or written pseudowords as compared to words, and is understood to reflect enhanced lexico-semantic memory search for pseudowords that have no lexical representation (e.g., [21-25]). Indeed, using magnetoencephalograhpy (MEG) and magnetic resonance imaging, generators for the scalp-recorded N400 have been localized among others in the left temporal lobe, comprising brain areas supporting long-term memory functions [26,27].

However, it appears as if ERP effects in the time course of the N400 component reflect several temporally and cognitively distinct processes [28]. In sentence context, patterns of N400 amplitude change prior to and after the minimum duration required to identify a word [29-32]. Even N400 amplitudes elicited by single words and pseudowords diverge as a function of the amount of information provided by the temporally unfolding speech signal [33]. Words and pseudowords do not differ for the initial N400 component, presumably because the speech signal activates several alternatives in the listeners' mental lexicon. Different N400 patterns occur as soon as the signal discriminates a pseudoword from all other entries in a given language (deviation point). Therefore, only the initial part of the N400 pseudoword effect might be related to normal fast word processing, whereas the later sustained negativity for pseudowords might reflect subsequent top-down guided evidence checking.

**Figure 1:**
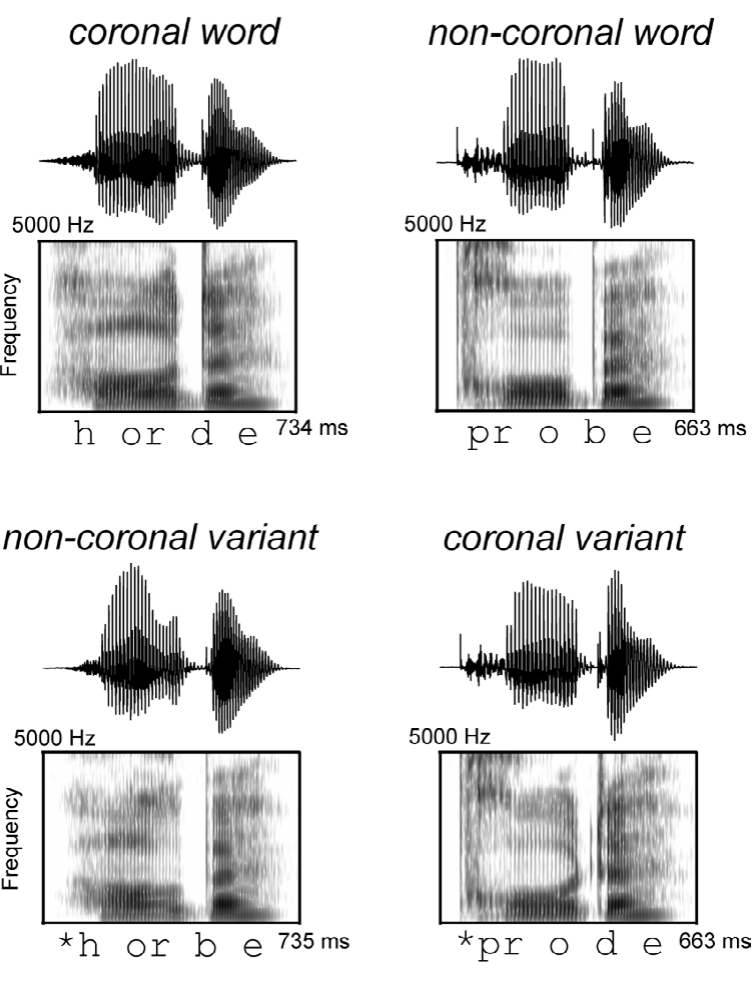
**Experimental Manipulation**. Examples of words (Above) and pseudoword variants (Below) are illustrated by respective speech sounds (Upper) and spectropraphic displays (Lower). Left: example for a presented coronal word (*Horde *[Engl. horde]) and its non-coronal variant (*Horbe). Right: Example for a presented non-coronal word (*Probe *[Engl. test]) and its coronal variant (*Prode).

In this study we presented spoken words and pseudoword variants that differed only in their medial consonant, which either had a coronal PLACE (/d/, /t/, /n/), or a non-coronal PLACE (LABIAL: /b/, /p/, /m/; DORSAL: /g/, /k/; see Figure 1 for an illustration of example stimuli). According to the FUL model lexical representations of words with word medial coronals like *Horde *('horde') have no word medial place represented in the lexicon. A non-coronal variant like *Horbe cannot mismatch this empty PLACE slot and therefore activates *Horde*. Although CORONAL PLACE is not represented in the lexicon, this feature can be perceived in the signal and can be used for lexical mapping. For example, a coronal pseudoword variant like *Prode mismatches the specified [LABIAL] PLACE of the German word *Probe *'test' and therefore cannot activate this word. If the assumption of underspecification holds, lexico-semantic memory search processes would be successful when a non-coronal variant is presented (first activating the corresponding coronal target word), but not when a coronal variant is presented (immediate correct rejection as a non-existing lexical item). Thus, we expect an asymmetry at least for the initial N400 pseudoword effect, which most likely is related to lexico-semantic processing. Related to the point in time where pseudowords diverge from their respective words (deviation points) initial N400 should be reduced for non-coronal variants as compared to coronal variants.

## Method

### Participants

Sixteen (eight females, eight males) undergraduate students from the University of Konstanz participated in the experiment. All participants were native speakers of German with no discernible uncorrected deficits in hearing. Participants were paid for their participation or received course credit points. Only right-handers were included, as ascertained by the Edinburgh Handedness Questionnaire [34].

### Stimuli

Ninety familiar German disyllabic nouns, with a medial stop or nasal consonant, and with stress on the first syllable were selected. Half of the words had a coronal consonant and the other half a non-coronal consonant as onset of the second syllable. A pseudoword with the opposite medial PLACE feature was formed for each word. Frequency of coronal and non-coronal words was matched using an on-line dictionary of German [35].

As neighborhood density is known to modify N400 amplitudes [36], we determined phonological neighbors of the words and pseudowords by using the CELEX database [37]. A neighbor is any word which differs from the stimulus by replacing, deleting or adding one phoneme in any position (cf. [38]). Only monomorphemic, disyllabic neighbors with stress on the first syllable were included. A two-way ANOVA with repeated measures factors *Lexical Status *(words vs. pseudoword variants), and *Coronality *(coronal words, and their variants vs. non-coronal words and their variants) only revealed a significant effect of *Lexical Status *[F(1,44) = 32.41, p < .001]. Words had on average 3.4 (S [tandard] D [eviation] 2.6) neighbors, pseudoword variants had 2.2 (SD = 1.8) neighbors. Neighborhood density did not differ for coronal words (M [ean] = 3.4, SD = 2.3) and non-coronal words (M = 3.4, SD = 2.8) [t(44) = 0.01, n.s.], nor for coronal pseudowords (M = 2.4, SD = 2.0) and non-coronal pseudowords (M = 2.0, SD = 1.6) [t(44) = 0.84, n.s.].

All words and pseudowords were spoken by a male native speaker of German in a sound attenuating chamber. The speaker was naïve with respect to the experimental manipulation and the hypotheses of the study. He read the stimuli in a fluent style from lists in which a word was immediately followed by the respective pseudoword variant. The speaker was instructed to pronounce the variants in the same way as the words. Stimuli were recorded on a DAT recorder at a sampling rate of 44.1 kHz using a high quality microphone. The recordings were then transferred to a computer, the volume equalized, and edited into individual tokens using the Cooledit 2000 waveform manipulation software package. Where possible, single periods of voiced speech sounds were deleted from either the word or the variant to equalize the duration of the stimuli. This resulted in the same mean length of coronal words and their non-coronal pseudoword variants (M = 678 ms, SD = 97 for both), and of the same mean length for non-coronal words and their coronal pseudoword variants (702 ms, SD = 102 for both).

Uniqueness points of the words and deviation points of the pseudowords were established using the CELEX lexical database [37]. Scanning from left to right, we determined the first phoneme of the words that made them unique with respect to all other entries in the CELEX database, and also the first phoneme of the pseudowords that made them distinguishable from any monomorphemic, disyllabic, first-syllable stressed word in the database. For 40 non-coronal words, 40 coronal words, 41 non-coronal pseudowords, and 40 coronal pseudowords this uniqueness or deviation point was the phoneme for which the place of articulation was manipulated. For the remaining 4 to 5 stimuli per group the uniqueness/deviation phoneme appeared later in the word. Next, the onset of the deviating phonemes, which was the onset of the closure period for plosives and the onset of the first period for nasals, was determined in the acoustic signal. Mean uniqueness points were 335 ms (SD = 107) for coronal words and 366 ms (SD = 104) for non-coronal words. Mean deviation points were 355 ms (SD = 98) for coronal pseudoword variants and 322 ms (SD = 104) for non-coronal pseudoword variants. These uniqueness and deviation points were used to adjust the ERP and behavioral data.

### Procedure

Sintered silver-silver chloride electrodes were held in place on the scalp with an elastic cap (EASY Cap). Scalp locations included 62 standard International 10–10 system locations. Two additional electrodes to control for eye movements were placed below both eyes. All electrodes were online referenced to Cz. The data were re-referenced offline to the algebraic average of the left and the right mastoids. All electrode impedances were less than 5 KΩ. The electroencephalogram (EEG) was recorded with a sampling rate of 250 Hz.

Subjects sat in a sound attenuated booth and made speeded lexical decisions to stimuli presented at a comfortable listening level (Sony loudspeakers). A trial started with a fixation point presented in the center of a computer screen. 200 ms after the onset of the fixation point a spoken stimulus was presented. The fixation point remained throughout the spoken stimulus and was terminated with the subject's response. Subjects were told not to blink and to look at the fixation point as long as it appeared on the screen. Following the fixation point there was a 1500 ms blank screen intertrial-interval. Subjects were told they could blink during this interval. Half the subjects made yes-responses with the thumb of their left hand and no-responses with the thumb of their right hand. For the remaining subjects, the response hands were reversed. Speed and accuracy were stressed equally. Subjects were given a break after half of the trials.

### Data analysis

**Figure 3:**
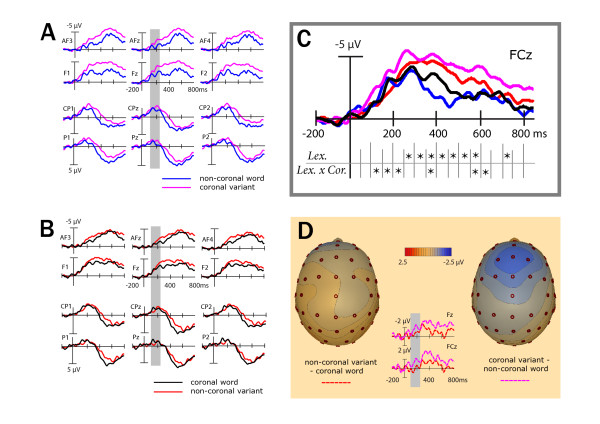
**Neurophysiological results time-locked to uniqueness and deviation points**. (A) plots the grand average ERPs for non-coronal words (blue lines) and coronal pseudoword variants (magenta lines) for selected electrode sites. (B) shows the grand average ERPs for coronal words (black lines) and non-coronal pseudoword variants (red lines) for selected electrode sites. (C) illustrates ERPs for all four experimental conditions for a representative electrode lead and summarizes outcomes of 50 ms time-steps analyses. Time windows yielding significant effects or trends (p < .10) of the factor *Lexical Status *(Lex.) or interactions of *Lexical Status *and *Coronality *(Lex. X Cor.) are indicated by an asterisk. (D) shows subtraction waves (variant-word) and respective scalp topography maps (180 ms after the uniqueness/deviation points). Across (A), (B) and (D) the part of the N400 pseudoword effect that is sensitive to different PLACE mismatches, is highlighted in light grey. For illustration purposes only ERPs were low-pass filtered (20 Hz).

Error rates were calculated for all stimuli. Reaction times and ERPs were only calculated for correctly responded trials. Eye blinks and movements were systematically recorded from each subject before the experimental task started. Characteristic scalp topographies of eye artifacts were corrected from the experimental data using Brain Electrical Source Analysis (BESA; ^® ^MEGIS Software GmbH). Word onset ERP data were quantified by calculating the mean amplitudes (relative to a 200 ms prestimulus baseline) in a latency window between 500 and 1000 ms (N400 pseudoword effect). ERPs time-locked to uniqueness and deviation points were quantified by calculating the mean amplitudes (relative to a 200 ms pre uniqueness and deviation point baseline) in two latency windows (early N400 pseudoword effect: 100–250 ms; late N400 pseudoword effect: 250–750 ms) according to 50 ms time-step analyses (see Figure 3C).

As there were virtually no interhemispheric effects, two regions of interest (ROIs) each including 20 lateral and midline electrode positions were defined. An anterior ROI included electrodes AF7, AF3, AFz, AF4, AF8, F5, F1, Fz, F2, F6, FC3, FC1, FCz, FC2, FC4, C5, C3, Cz, C4 and C6; a posterior ROI included electrodes CP5, CP3, CP1, CPz, CP2, CP4, CP6, P7, P3, P1, Pz, P2, P4, P8, T7, TP7, TP8, T8, PO1 and PO2. Two factors entered the repeated measures ANOVAs for behavioral data: *Lexical Status *(words vs. pseudoword variants), and *Coronality *(coronal words, and their variants vs. non-coronal words and their variants). An additional repeated measures factor *Region *(anterior vs. posterior electrode leads) was included in the ANOVA with the mean ERP amplitudes as the dependent variable. Scalp topographies were generated using BESA.

## Results

### Behavioral results

Mean reaction times from stimulus onset and from uniqueness/deviation points as well as error rates are shown in Table 1. Starting from stimulus onset subjects needed 976 ms to correctly identify words, and 1003 ms to correctly reject pseudoword variants. As shown by the main effect of *Lexical Status *[F(1,15) = 27.64, p < .001] this difference was statistically significant. A main effect of *Coronality *[F(1,15) = 17.84, p < .001] indicated that responses were faster for coronal words and their respective pseudoword variants than for non-coronal words and their pseudoword variants. This might well be caused by the longer duration of the former as compared to the latter stimuli (see Methods section). Factors *Lexical Status *and *Coronality *did not interact [F(1,15) = 0. 05, n.s.].

**Table 1:** Summarized in this table are mean reaction times (RT) in ms from stimulus onset and from uniqueness/deviation points (UP/DP), and error rates in percent for all conditions (with standard deviations).

	***RT***		***Error Rates***
	*Stimulus Onset*	*UP/DP*	
Noncoronal words	980 (98)	613 (97)	6.7 (4.3)
Coronal variant	1026 (125)	671 (125)	2.2 (2.1)
Coronal words	951 (96)	616 (95)	7.5 (4.8)
Non-coronal variant	1001 (107)	676 (107)	4.9 (3.1)

In order to control for different word lengths across both *Coronality *conditions, individual uniqueness points were subtracted from individual reaction times to words and deviation points were subtracted from reaction times to pseudowords. After this correction, only the main effects of *Lexical Status *remained significant [F(1,15) = 39.79, p < .001]. Words were identified 615 ms after their uniqueness point, pseudoword variants were identified 674 ms after their deviation points. The interaction of *Lexical Status *and *Coronality *remained to be not significant [F(1,15) = 0.03]. It took subjects the same time to reject coronal or non-coronal pseudowords after the appearance of their respective deviation points.

Analysis of error rates revealed that speeded responses to words were associated with lower accuracy. Subjects made 7% errors for words and 3.5% errors for pseudoword variants [F(1,15) = 13.51, p < .01]. Furthermore, a main effect of *Coronality *was observed for error rates [F(1,15) = 5.23 p < .05]. Subjects made more errors for coronal words and their variants (6.1%, SD = 4.2) than for non-coronal words and their variants (4.4%, SD = 4.0). No significant interactions of *Lexical Status *and *Coronality *were observed for reaction times or error rates [F(1,15) = 1.41, n.s.].

### ERPs time-locked to stimulus onset

**Figure 2:**
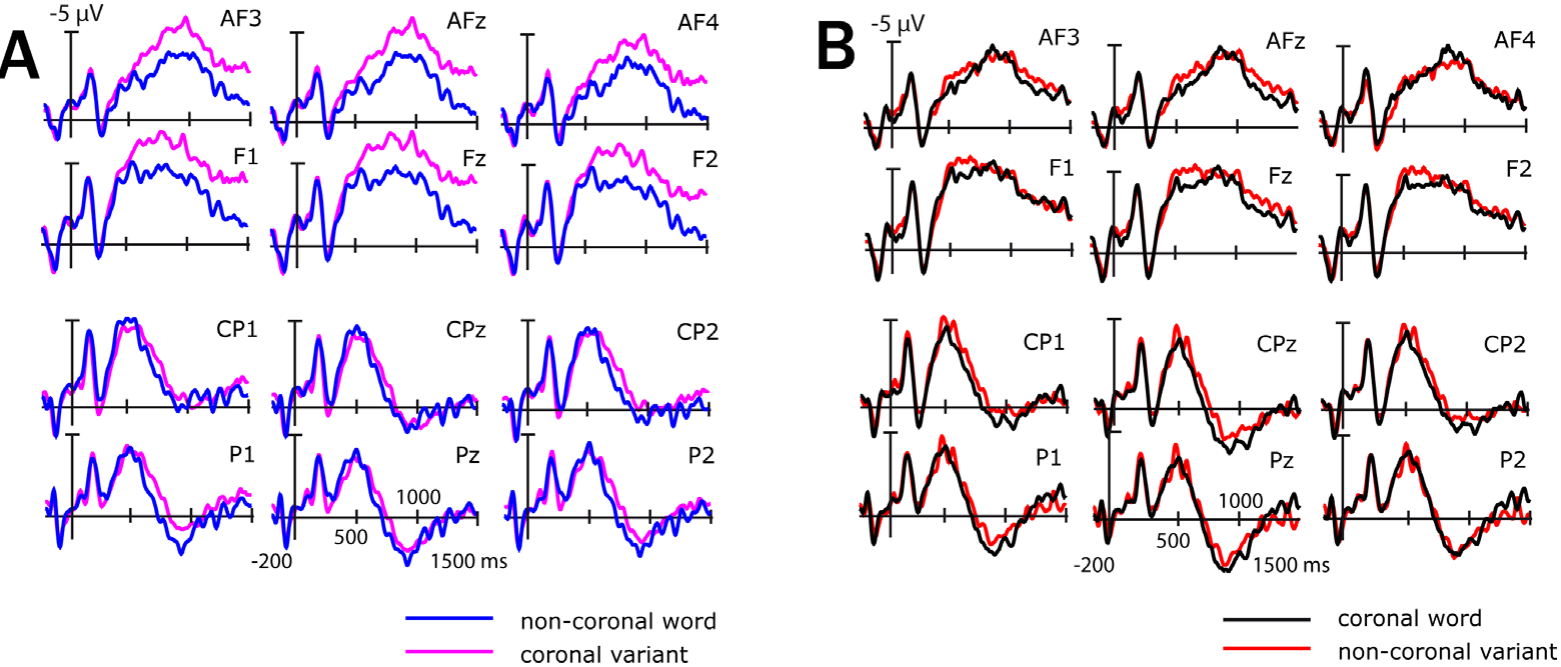
**Neurophysiological results time-locked to word onset**. (A) plots the grand average ERPs for non-coronal words (blue lines) and coronal pseudoword variants (magenta lines) for selected electrode sites. (B) shows the grand average ERPs for coronal words (black lines) and non-coronal pseudoword variants (red lines) for selected electrode sites. For illustration purposes only ERPs were low-pass filtered (20 Hz).

The ERP grand mean waveforms time-locked to the onsets of words and pseudoword variants for selected anterior and posterior electrode sites are plotted in Figure 2. For all the ERPs, the first visible component was a negative-going deflection peaking at 130 ms after stimulus onset (N1). This was followed by a positive deflection occurring at approximately 200 ms (P2). Starting at 300 ms a broad negativity was observed, which was enhanced for pseudoword variants as compared to words and is henceforth referred to as the N400 pseudoword effect. Additionally, a late positivity ranging between 600 and 1500 ms was observed for posterior electrode leads. A time window ranging from 500 to 1000 ms was used to further establish the N400 pseudoword effect time-locked to stimulus onset.

#### Time window 500 to 1000 ms

Significant main effects for the factors *Region *[F(1,15) = 46.46, p < .001] and *Lexical Status *[F(1,15) = 8.21, p = .01] were observed in the time window of the N400 pseudoword effect time-locked to stimulus onset. ERPs were more negative for frontal electrode leads than for posterior electrode leads, and more negative for pseudowords than for words. An interaction of the factors *Lexical Status *and *Coronality *did not reach significance [F(1,15) = 2.75, p = .11].

Overall, an N400 pseudoword effect could be established in the present experiment. However, temporal analysis of this effect has to consider different uniqueness and deviation points within and across conditions. Next we adjusted the N400 pseudoword effect to the point in time where differences between the words and their respective pseudoword variants occurred in the acoustic signal.

### ERPs time-locked to uniqueness and deviation points

The ERP grand mean waveforms time-locked to the uniqueness points of the words and to the deviation points of the pseudoword variants for selected anterior and posterior electrode sites are plotted in Figure 3. The ERPs zoom into the N400 effect and the posterior positivity. 50 ms time-steps analyses suggest an earlier differentiation of N400 effects for coronal and non-coronal pseudowords (see Figure 3C). A time window ranging from 100 to 250 ms was analyzed to investigate the early pseudoword N400 effect for ERPs adjusted to uniqueness and deviation points. The later N400 effect was analyzed in a time window ranging from 250 to 750 ms.

#### Time window 100 to 250 ms

Early N400 amplitudes showed a main effect of *Region *[F(1,15) = 8.46, p = .02], which did not interact with any other factor. ERPs were more negative over posterior than over anterior electrode leads. Crucially, an interaction of the factors *Lexical Status *and *Coronality *licensed differentiation of coronal and non-coronal pseudoword effects [F(1,15) = 6.22, p = .02]. Mean amplitudes for coronal pseudoword variants were more negative than mean amplitudes for their non-coronal base words [t(15) = 6.19, p = .02]. By contrast, ERPs for non-coronal variants did not differ from their base words in this initial part of the N400 pseudoword effect [t(15) = 0.26, n.s.]. Furthermore, a significant difference between both types of pseudoword variants [t(15) = 12.21, p < .01] but not between both types of words [t(15) = 0.23, n.s.] relates this early ERP deflection to mismatch detection in the case of coronal pseudowords.

#### Time window 250 to 750 ms

Significant main effects for the factors *Region *[F(1,15) = 98.42, p < .001] and *Lexical Status *[F(1,15) = 11.66, p < .01] were observed in the second time window of the ERPs time locked to uniqueness and deviation points. ERPs were more negative for frontal electrode leads and more positive for posterior electrode leads. No interaction effect reached significance revealing that both types of pseudoword variants were differentiated in a same way from their respective base words in the later time window of the N400 pseudoword effect.

## Discussion

This paper examined neurophysiological correlates of spoken word identification, by means of ERPs recorded during a lexical decision task. The question we asked was how the recognition system copes with pseudowords that differ only slightly from existing words, namely in word medial place of articulation (PLACE). Assuming an underspecified coronal PLACE, we predicted asymmetric effects for pseudowords being either coronal or non-coronal variants. In accordance with our hypotheses the N400 pseudoword effect starts earlier for coronal than for non-coronal pseudowords.

Our results support assumptions of the FUL model of spoken word recognition [2-4]. In this model coronal words are proposed to have an empty feature value slot for PLACE in the mental lexicon. Even though a LABIAL or DORSAL PLACE is extracted from non-coronal pseudowords they can activate lexical representations of coronal words (i.e. *Horbe can activate *Horde*). Hence, non-coronal pseudowords ought to behave like words at the initial activation period. The opposite hypothesis prevails for non-coronal words. FUL assumes that non-coronal entries have PLACE specified. Coronal variants, for which a CORONAL PLACE is extracted from the signal, cannot activate non-coronal words (i.e., *Prode cannot activate *Probe*). That is, coronal variants should be regarded as nonwords earlier than non-coronal variants. In line with these assumptions, coronal variants elicited an earlier N400 pseudoword effect than non-coronal variants.

The fact that it took subjects the same time to reject both types of pseudoword variants, together with the finding that pseudoword rejection took longer than word acceptance, point to a process that detects pseudowords and guides responses in the lexical decision task independently of initially asymmetric lexical activation. The present ERP results also provide evidence for an additional process in the lexical decision task. Both types of pseudoword variants elicited enhanced negativity in a later time window of the N400 pseudoword effect. The FUL model may account for this later ERP effect and for the behavioral results by assuming phonological parsing mechanisms that are independent of initial lexical activation [4]. One could surmise that a post-lexical mechanism rejects both coronal and non-coronal pseudoword variants. Whether this evidence checking operation is mandatory in spoken word recognition or whether it is especially recruited to solve the lexical decision task has to be clarified in future research.

A separation of lexical and post-lexical processing in the N400 time window has already been suggested in previous ERP research on speech recognition. Specifically it has been argued that pseudowords undergo some additional post-lexical processing that is reflected in the sustained N400 pseudoword effect [33,39]. Similarly it has been shown that negative going ERP effects in spoken sentence comprehension are at least two-fold [29-32]. An early negativity starting 150 ms after word onset is equally reduced for words that fit into the semantic context of a spoken sentence (e.g., *She illuminated the dark room with her ****candle***.), as well as for words that semantically do not fit into a spoken sentence context, but match the initial phonemes of the fitting word (e.g., *She illuminated the dark room with her ****candy***.). Only later, starting at 300 ms, semantically acceptable words diverge from those that had initial phoneme overlap. Hagoort and coworkers argue that the early interaction of word-form and content information is related to ongoing lexical selection, whereas the later N400 effect is associated to semantic integration of selected candidates.

In sum, we separate an early from a later part of the N400 pseudoword effect. Integrating current and previous ERP findings we conclude that the initial part of the N400 pseudoword effect closely relates to lexical activation pattern in spoken word recognition. We argue that the asymmetry of this effect reflects that pseudoword variants can only activate underspecified cortical word form representations in the listeners' mental lexicon. The early onset of the N400 pseudoword effect (100 ms after the deviation point) might reflect that PLACE information is already exploited at the vowel that precedes the deviating phoneme [40]. Crucially, however, the early onset of the N400 pseudoword effect for coronal variants suggests that the CORONAL PLACE is immediately used to prevent lexical activation of non-coronal words.

Our findings add important arguments on theoretical accounts to spoken word recognition. First, the early asymmetric N400 pseudoword effect challenges context based models of assimilated speech recognition [5,6,8-10]. The fact that the pseudoword N400 effect for coronal variants starts 100 ms after the onset of the coronal speech sound reveals that initial lexical activation does not wait until contextual information is available. Second, asymmetric lexical activation is observed for the first time in pseudowords created by replacing medial consonants in monomorphemic words. Such pseudowords cannot be the result of regular assimilation patterns with final consonants like green bag > *gree [m] bag, and thus listeners are never exposed to such variants. That is, there could be no variant exemplars stored for medial stops and nasals, and therefore an exemplar based model (e.g. [41]) cannot account for the observed asymmetry. Third, asymmetric pseudoword effects for coronal and non-coronal pseudowords cannot be captured by models of spoken word recognition that assume that all segments, usually phonemes, equally contribute to lexical activation (e.g., [42-44]). The fact that we have observed different ERP effects for coronal and non-coronal pseudowords, which start immediately after the deviation is present in the signal and which are in the direction proposed by the FUL model, supports our assumption that underspecification is a basic principle of the functional organization of the mental lexicon.

## Conclusion

The FUL model of spoken word recognition [2-4] provides excellent theoretical underpinning for the discovery of neurophysiological mechanisms underlying human speech comprehension. Former studies showed that phonological features are extracted early from the signal and that cortical representations of speech are based on those features [45-47]. Furthermore, the asymmetric mapping of features onto specified and underspecified cortical vowel representations has been established in a previous ERP study that used an MMN paradigm [18]. The present findings are in accordance with this line of neurophysiological evidence. Using a different ERP component and more complex linguistic stimuli, the present study supports the notion of specified and underspecified cortical word form representations and an asymmetric mapping of features extracted from word medial positions onto those lexical representations.

## Authors' contributions

CF designed and conducted the study, analyzed the ERP data, and wrote the initial drafts. CE contributed to the ERP analysis. AL provided the theoretical background. All authors contributed to the selection of the stimulus materials, experimental design and worked on the manuscript.
